# Association between college students 'physical fitness index and negative emotional symptoms: an empirical study based on college students in Chongqing City, China

**DOI:** 10.3389/fpsyg.2026.1927005

**Published:** 2026-09-24

**Authors:** Shijun Tan, Caifeng Li

**Affiliations:** College of Physical Education and Health Management, Chongqing University of Education, Chongqing, China

**Keywords:** anxiety, college students, depression, negative emotional symptoms, physical fitness index, stress

## Abstract

**Objectives:**

To investigate the independent associations between the physical fitness index (PFI), as well as individual physical fitness metrics, and negative emotional symptoms (stress, anxiety, and depression) among college students; and to analyze gender-specific variations in these associations, thereby providing empirical, fitness-based evidence for mental health interventions in higher education institutions.

**Methods:**

Utilizing a cross-sectional design, this study selected 2,190 college students from 73 universities across Chongqing via stratified cluster sampling. A standardized physical fitness test battery—comprising vital capacity, the 50-meter sprint, the 800/1000-meter run, the sit-and-reach test, and pull-ups/sit-ups—was administered to calculate the comprehensive PFI. Negative emotional symptoms were assessed using the 21-item Depression Anxiety Stress Scales (DASS-21). Multivariable Logistic regression models were employed to evaluate the independent associations between physical fitness and negative emotional symptoms, after comprehensively adjusting for moderate-to-vigorous physical activity (MVPA), muscle-strengthening exercise (MSE), screen time, sedentary duration, and sociodemographic variables.

**Results:**

The detection rates of stress, anxiety, and depression symptoms were 68.08%, 67.67%, and 12.37%, respectively. In conventional categorical models, moderate and high PFI were associated with lower odds of all three outcomes in the pooled sample, although several high-PFI estimates were extremely small. Firth sensitivity analyses supported inverse associations for high PFI across outcomes and sexes; however, moderate PFI was not statistically associated with stress in female students or anxiety in male students. Continuous PFI analyses showed lower odds of each outcome per 1-SD increase in the pooled and sex-stratified samples (all *P* < 0.001). These patterns were broadly consistent with the individual-component analyses, but the cross-sectional design precludes causal interpretation.

**Conclusions:**

Higher PFI was independently associated with lower odds of negative emotional symptoms among college students. The consistency of direction across categorical, Firth-penalized, and continuous-PFI models supports the robustness of the association, but the extreme categorical estimates, model-calibration findings, sample-relative cutoffs, and cross-sectional design require cautious interpretation. Longitudinal and intervention studies are needed before PFI is used as a clinical or campus screening threshold.

## 1 Introduction

The undergraduate stage represents a critical window of vulnerability for the onset and escalation of mental health disorders (Zhang et al., 2025a). Epidemiological evidence indicates that the global pooled prevalence rates of depressive and anxiety symptoms among college students are as high as 33.6% and 39.0%, respectively—levels that are significantly elevated compared to their non-student peers (Li et al., 2022). Negative emotional symptoms, including depression, anxiety, and stress, not only severely impair academic performance, social adaptation, and overall quality of life among undergraduate students, but also precipitate long-term psychosomatic health risks (Zada et al., 2021; Kusumaningsih, 2016; McCloughen et al., 2012), imposing a profound clinical and economic burden on global public health systems (Collaborators, 2022). Consequently, identifying modifiable, actionable factors to facilitate early prevention of psychological distress has emerged as a paramount objective for health promotion initiatives within higher education worldwide.

Among a myriad of non-pharmacological interventions, physical activity and physical fitness have garnered substantial academic attention due to their multi-systemic health benefits (Elbe et al., 2019). Cumulative evidence suggests that habitual physical activity is inversely associated with the risk of depressive, anxious, and stress symptoms, and structured exercise interventions have been empirically validated as effective modalities for mitigating mental health problems in college populations (Singh et al., 2023). Recently, a paradigm shift has occurred in the literature; research focus is progressively transitioning from subjective, self-reported metrics—such as exercise frequency and duration—toward the objective and granular evaluation of multidimensional physical fitness components, including cardiorespiratory fitness, muscular strength, speed, endurance, and flexibility (Banyard et al., 2025). Rather than serving as mere markers of physical capacity, these objective fitness indices play a pivotal role in constructing a physiological buffer against psychological stressors. This protective mechanism is hypothesized to operate by reshaping the neuroendocrine microenvironment, optimizing sleep architecture, and enhancing physical self-efficacy (Lubans et al., 2016).

However, the existing literature remains predominantly fragmented, focusing either on isolated physical activity behaviors or on individual fitness metrics in relation to mental health, while largely overlooking the holistic impact of comprehensive, global fitness levels on negative emotional symptoms. The Physical Fitness Index (PFI) comprehensively integrates core somatic parameters—such as vital capacity, speed, strength, endurance, and flexibility—thereby offering a more holistic and accurate depiction of an individual's true physiological fitness homeostasis than any single metric in isolation. To bridge this knowledge gap, the present study utilizes a large-scale, empirical sample of Chinese university students from Chongqing to systematically examine the independent associations of both global PFI and its specific sub-dimensions with the risk of depressive, anxious, and stress symptom detection. Furthermore, we aim to elucidate the gender-specific variations inherent in these associations, with the ultimate goal of providing a robust, empirical foundation for designing targeted, synergistic physical-mental health interventions in university settings.

## 2 Methods

### 2.1 Participants and sampling

A multi-stage stratified random sampling matrix was utilized to recruit participants from the official directory of higher education institutions in Chongqing, China. The field physical fitness testing and questionnaire surveys were concurrently administered between December 2025 and January 2026.

The sampling framework was operationalized through the following sequential stages:

Stage 1: A simple random sampling method was deployed to select 73 universities across Chongqing Municipality.

Stage 2: Within each selected university, one secondary college/department was randomly chosen.

Stage 3: From the designated secondary colleges, undergraduate students in their sophomore year (Grade 2) were targeted. Gender-stratified random sampling was subsequently implemented within this cohort, yielding a final allocation of 16 male and 16 female students per university.

Prior to data collection, all candidates were fully informed of the research objectives, and voluntary written informed consent was obtained. A total of 2,336 questionnaires were initially distributed. After applying strict exclusion criteria—which included eliminating protocols with incomplete questionnaire responses, missing objective physical fitness test data, or logical inconsistencies—a final sample of 2,190 valid datasets was retained, representing a high response efficiency of 93.75%. The final cohort comprised 1,096 male students (50.05%) and 1,094 female students (49.95%). All protocols were conducted in strict accordance with institutional ethical guidelines, and all individual data were utilized exclusively for anonymized academic research.

### 2.2 Methodology

#### 2.2.1 Sociodemographic information

Sociodemographic characteristics were systematically collected using a structured inventory, which included gender, age, academic grade, affiliated college, and academic major.

#### 2.2.2 Physical fitness assessment

Objective physical fitness data were obtained through supervised, on-site field testing (Ministry of Education of the People's Republic of China, 2014), strictly adhering to the protocols and operational guidelines outlined in the National Student Physical Health Standard (2014 Revision) (hereafter referred to as the Standard). The assessment battery covered three domains—physiological function, physical capacity, and somatic morphology—whereas the analytic PFI used in the present study was restricted to indicators of physiological function and physical capacity. Somatic morphology was assessed separately using BMI.

Physiological Function: Assessed via vital capacity.

Physical Capacity: Evaluated across multiple sub-dimensions, including flexibility (the sit-and-reach test), speed (the 50-meter sprint), lower-limb muscular power (the standing long jump), muscular strength/endurance (pull-ups for male students; 1-min sit-ups for female students), and cardiorespiratory endurance (the 1,000-meter run for males; the 800-meter run for females).

Somatic Morphology: Body Mass Index (BMI) was calculated using the standard formula:


$$\begin{array}{l} BMI=weight\left(kg\right)/height^{2}{\left(m\right)}^{2} \end{array}$$

Participants were subsequently classified into four distinct nutritional status categories according to the weight-stratified scoring matrix of the Standard: underweight, normal weight, overweight, and obese. Although BMI is a component of the National Student Physical Health Standard, it was not incorporated into the PFI for two reasons. First, BMI represents body size and nutritional status rather than functional physical performance, and its interpretation is non-monotonic because both low and high values may indicate unfavorable nutritional status. In contrast, the six PFI components could be directionally transformed so that higher values consistently represented better functional fitness. Second, BMI classification was retained as a separate covariate in the regression models; incorporating BMI into the PFI while simultaneously adjusting for BMI would introduce part–whole overlap and complicate interpretation. Therefore, BMI was analyzed separately rather than aggregated into the PFI.

Accordingly, to establish a uniform, dimensionless metric, the raw scores of the six physiological-function and physical-capacity indicators were standardized using Z-scores according to the following formula:


$$z\;=\;\frac{x\;-\;\mu }{\sigma }$$

where *x* represents the individual raw score of a specific test metric, μ represents the sex-specific sample mean, and σ denotes the corresponding standard deviation.

Subsequently, the individual *Z*-scores were aggregated to composite the global Physical Fitness Index (PFI) (Li et al., 2020). Given that the 50-meter sprint and the 1,000-meter/800-meter endurance runs are time-dependent metrics (where higher values denote lower physical performance), their standardized scores were reverse-scored (*multiplied by*−1) prior to composite calculation. The final PFI was operationalized using the following mathematical expression:


$$\begin{array}{l} PI=Z_{vital\;capacity}\;-\;Z_{50m\;sprint}\;+\;Z_{standing\;long\;jump}\\ +\;Z_{muscular\;strength}\;-\;Z_{endurance\;run\;}\;+\;Z_{sit\;-\;and\;-\;reach} \end{array}$$

A higher PFI score represents better comprehensive physical fitness. To account for sex-specific performance distributions, PFI categories were defined separately within female and male students using the sex-specific sample percentiles (Yang and Ma, 2024). Low PFI was <P25, moderate PFI was P25–P75 (inclusive), and high PFI was >P75.

#### 2.2.3 Assessment of depression, anxiety, and stress

Negative emotional symptoms among college students were evaluated using the 21-item Depression Anxiety Stress Scales (DASS-21), originally developed by Lovibond and Lovibond (1995) and psychometrically validated for Chinese populations by Gong et al. (2010). This instrument has demonstrated robust reliability and validity across diverse demographic cohorts.

The DASS-21 is structured into three distinct subscales—depression, anxiety, and stress—with each subscale comprising 7 items (21 items in total). Responses for each item are scored on a 4-point Likert scale ranging from 0 (did not apply to me at all) to 3 (applied to me very much, or most of the time), based on the participants' subjective experiences over the preceding week. The raw score for each subscale is calculated by summing the scores of its 7 constituent items, which is subsequently multiplied by 2 to yield a final subscale score ranging from 0 to 42.

According to the scoring manual, severity categories are operationalized based on the following specific cut-off thresholds:

Stress: Normal (0 ≤ score ≤ 14), mild (15 ≤ score ≤ 18), moderate (19 ≤ score ≤ 25), severe (26 ≤ score ≤ 33), and extremely severe (≥ 34).

Anxiety: Normal (0 ≤ score ≤ 7, mild (8 ≤ score ≤ 9), moderate (10 ≤ score ≤ 14 ), severe (15 ≤ score ≤ 19), and extremely severe (≥20).

Depression: Normal (0 ≤ score ≤ 9, mild (10 ≤ score ≤ 13), moderate (14 ≤ score <20), severe (21 <score <27), and extremely severe (≥ 28).

In the present dataset, Cronbach's α was 0.931 for the total DASS-21 and 0.853, 0.810, and 0.844 for the stress, anxiety, and depression subscales, respectively. For prevalence and regression analyses, each subscale was dichotomized as 0 = normal and 1 = mild-or-higher symptoms, using thresholds of ≥15 for stress, ≥8 for anxiety, and ≥10 for depression (Zhang et al., 2025b).

#### 2.2.4 Covariates assessment

Covariates adjusted in the statistical models comprised moderate-to-vigorous physical activity (MVPA), muscle-strengthening exercise (MSE), autonomous exercise, organized sports participation, daily screen time, and daily sedentary duration.

MVPA was operationalized as the total number of days per week on which participants accumulated at least 60 min of MVPA. This was evaluated via two items:

“During the past week, on how many days from Monday to Friday did you accumulate at least 60 min of MVPA?”

(options ranging from 0 to 5 days) and “During the past week, on how many days from Saturday to Sunday did you accumulate at least 60 min of MVPA?” (options ranging from 0 to 2 days). The raw scores from weekdays and weekends were aggregated to yield a cumulative weekly MVPA score ranging from 0 to 7 days. This psychometric assessment protocol has demonstrated excellent test-retest reliability (Intraclass Correlation Coefficient, *ICC* = 0.82 (Liu et al., 2010) and desirable criterion validity against accelerometer-measured metrics (*r* = 0.40) (Prochaska et al., 2001) within Chinese youth populations.

MSE was evaluated based on the weekly frequency of engagement in muscle-strengthening activities. Participants responded to the item:

“During the past week, on how many days from Monday to Sunday did you engage in MSE, such as push-ups, sit-ups, or weightlifting?”

(options ranging from 0 to 7 days). This single-item instrument possesses acceptable reliability (*k*>0.55) (Morrow Jr et al., 2013) and has been widely implemented in national youth health behavior surveillance systems across the United States and Canada (Merlo, 2020; Harvey et al., 2017).

Autonomous exercise was assessed by the question:

“Over the past year, excluding regular physical education (PE) classes, how many times per week did you engage in autonomous/unstructured exercise?”

with responses scored on an ordinal scale: 0, 1, 2, 3, 4, and ≧5 times/week. Organized sports participation was defined as involvement across four sub-categories: school varsity sports teams, intramural sports clubs, after-school sports competitions, and off-campus commercial sports training. The item was framed as:

“Over the past year, excluding regular PE classes, how many times per week did you participate in school varsity sports teams / intramural sports clubs / after-school sports competitions / off-campus sports training?”

utilizing the identical response options of 0, 1, 2, 3, 4, and ≧5 times/week. In subsequent multivariable analyses, these frequencies were treated as categorical variables.

Daily screen time and daily sedentary duration were collected through validated student self-reports. Specifically, screen time was defined as the cumulative daily duration dedicated to screen-based devices (e.g., smartphones, computers, tablets, and televisions), while sedentary duration captured the total daily time spent in a sitting or low-energy-expenditure posture. Both lifestyle variables were classified into five distinct categorical levels: virtually none, <1 h, 1–2 h, 2–3 h, and > 3 h.

### 2.3 Quality control

To guarantee the precision, validity, and reliability of the survey responses and physical fitness metrics, a rigorous multi-stage quality assurance protocol was enforced throughout the study lifecycle.

#### 2.3.1 Pre-implementation phase

Prior to data collection, all field investigators and test administrators underwent standardized, centralized training. This training synchronized the operational workflows, standardized questionnaire instruction scripts, and calibrated the physical fitness testing criteria. Simultaneously, all digital physical fitness testing apparatuses and anthropometric instruments were rigorously inspected, cross-calibrated, and verified to eliminate inter-device variability and ensure optimal functional alignment.

#### 2.3.2 Implementation phase

The questionnaire survey was administered in a supervised, group-based, on-site setting. Investigators delivered unified, standardized explanations regarding the response format. During the sessions, investigators provided real-time clarification and active oversight to prevent missing data, erroneous responses, or casual, non-serious completions.

#### 2.3.3 Data clean-up and verification phase

In the data curation stage, a strict completion-time filter was operationalized; questionnaires completed in less than 5 min (<5 min) were flagged as logical anomalies and excluded from the analysis. Subsequently, an algorithmic data-linkage and consistency check was executed across the psychometric datasets and objective physical fitness matrices, systematically identifying and eliminating protocols with missing data, structural logical contradictions, or incomplete physical health metrics. Finally, all datasets finalized for statistical analyses underwent independent double-entry verification and cross-checking by two researchers to guarantee absolute fidelity, completeness, and academic integrity.

### 2.4 Statistical analysis

Data curation and descriptive analyses were conducted using Microsoft Excel 2007 and SPSS version 24.0 (IBM Corp., Armonk, NY, USA). Recalculation, penalized regression, and sensitivity analyses were conducted in R version 4.6.1 (R Foundation for Statistical Computing, Vienna, Austria).

Categorical variables were summarized as n (%) and compared using Pearson's χ^2^ tests. Binary outcomes were coded as 0 = absence and 1 = presence of mild-or-higher stress, anxiety, or depressive symptoms. Two complementary model families were specified. In Model A (Table 1), the six sex-standardized component scores—vital capacity, 50-m sprint, standing long jump, muscular strength, endurance run, and sit-and-reach—were entered jointly to estimate component-specific associations. In Model B (Table 2), the categorical global PFI was entered with low PFI as the reference group. Because PFI is constructed from the six component scores, PFI and its components were not entered in the same model.

**Table 1:** Multivariable logistic regression of individual physical-fitness components and DASS-21 symptom outcomes.

Gender	Independent Variable	Pressure			Anxiety			Depression		
		β	OR (95%CI)	*p*	β	OR (95%CI)	*P*	β	OR (95%CI)	*p*	
Female (*n* = 1,094)	Z_vital capacity_	−0.71	0.49(0.41–0.59)	0.00	−0.45	0.64(0.55–0.74)	0.00	−0.20	0.82(0.67–1.00)	0.05
	Z_50m sprint_	0.71	2.03(1.41–2.92)	0.00	0.37	1.45(1.11–1.90)	0.01	0.22	1.25(0.99–1.57)	0.06
	Z_standing long jump_	−0.78	0.46(0.34–0.61)	0.00	−0.31	0.74(0.61–0.89)	0.00	0.08	1.08(0.85–1.38)	0.51
	Z_muscular strength_	−0.42	0.66(0.54–0.79)	0.00	−0.34	0.71(0.60–0.83)	0.00	−0.11	0.90(0.73–1.11)	0.31
	Z_endurance run_	0.49	1.64(1.30–2.06)	0.00	0.31	1.37(1.14–1.64)	0.00	0.13	1.14(0.91–1.42)	0.26
	Z_sit-and-reach_	−0.60	0.55(0.46–0.66)	0.00	−0.17	0.85(0.73–0.98)	0.03	−0.08	0.92(0.76–1.12)	0.40
Male (*n* = 1,096)	Z_vital capacity_	−0.36	0.69(0.59–0.82)	0.00	−0.48	0.62(0.52–0.75)	0.00	−0.15	0.86(0.72–1.03)	0.10
	Z_50m sprint_	0.66	1.93(1.46–2.58)	0.00	0.61	1.84(1.32–2.56)	0.00	0.30	1.35(1.06–1.71)	0.01
	Z_standing long jump_	−0.07	0.93(0.75–1.16)	0.52	−0.56	0.57(0.41–0.79)	0.00	−0.07	0.93(0.77–1.13)	0.48
	Z_muscular strength_	−0.53	0.59(0.49–0.70)	0.00	−0.64	0.53(0.44–0.64)	0.00	−0.22	0.80(0.62–1.04)	0.10
	Z_endurance run_	0.61	1.84(1.49–2.26)	0.00	0.64	1.90(1.52–2.37)	0.00	0.34	1.40(1.14–1.73)	0.00
	Z_sit-and-reach_	−0.68	0.51(0.42–0.61)	0.00	−0.63	0.53(0.44–0.65)	0.00	−0.17	0.85(0.71–1.02)	0.08

**Table 2:** Multivariable conventional logistic regression of categorical PFI and DASS-21 symptom outcomes.

Gender	PFI	Stress			Anxiety			Depression		
	β	OR (95% CI)	*P*	β	OR (95% CI)	*P*	β	OR (95% CI)	*P*
Female (*n* = 1,094)	Low	—	1.000 Reference	—	—	1.000 Reference	—	—	1.000 Reference	—
	Moderate	−0.498	0.608 (0.359–1.031)	0.065	−0.405	0.667 (0.451–0.986)	0.042	−0.696	0.499 (0.321–0.774)	0.002
	High	−6.192	0.002 (0.001–0.005)	<0.001	−3.017	0.049 (0.031–0.077)	<0.001	−0.942	0.390 (0.222–0.684)	0.001
Male (*n* = 1,096)	Low	—	1.000 Reference	—	—	1.000 Reference	—	—	1.000 Reference	—
	Moderate	−1.990	0.137 (0.068–0.273)	<0.001	−0.245	0.782 (0.442–1.386)	0.400	−1.499	0.223 (0.146–0.342)	<0.001
	High	−6.109	0.002 (0.001–0.005)	<0.001	−5.014	0.007 (0.003–0.013)	<0.001	−2.093	0.123 (0.064–0.237)	<0.001
Overall (*n* = 2,190)	Low	—	1.000 Reference	—	—	1.000 Reference	—	—	1.000 Reference	—
	Moderate	−1.063	0.346 (0.230–0.518)	<0.001	−0.355	0.701 (0.511–0.962)	0.028	−1.113	0.329 (0.245–0.441)	<0.001
	High	−5.451	0.004 (0.003–0.007)	<0.001	−3.774	0.023 (0.016–0.033)	<0.001	−1.509	0.221 (0.146–0.334)	<0.001

Because high-PFI outcome cells were sparse and several ORs were extremely small, inference should be considered together with Supplementary Tables 1–4.

All overall models adjusted for sex, BMI category, weekly MVPA days (Larouche et al., 2014), weekly MSE days (Guo and Rao, 2021), autonomous exercise frequency, organized sports participation, daily screen-time category, and daily sedentary-time category. Sex-stratified models omitted sex. MVPA and MSE were modeled linearly per additional day/week; the remaining behavioral variables and BMI were categorical. Reference levels were female sex, normal BMI, 0 autonomous exercise sessions/week, 0 organized sports sessions/week, no screen time, and no sedentary time. All tests were two-sided, with *P* < 0.05 considered statistically significant.

To evaluate the extremely small odds ratios in the categorical PFI analyses, positive and negative outcome counts were cross-tabulated by PFI category (Supplementary Table 1) and model convergence and sparse-data patterns were reviewed. Firth penalized logistic regression with Jeffreys-prior penalization was then fitted as a sensitivity analysis; profile penalized-likelihood 95% confidence intervals and penalized likelihood-ratio *P* values are reported in Supplementary Table 2. In a second sensitivity analysis, PFI was standardized using the overall sample SD (3.5003 units) and modeled continuously per 1-SD increase using conventional logistic regression (Supplementary Table 3).

Conventional categorical-PFI models were assessed for convergence, calibration using the 10-group Hosmer–Lemeshow test, explanatory performance using Nagelkerke *R*^2^, and multicollinearity using the adjusted generalized variance inflation factor [GVIF^∧^(1/(2 × df))]. Diagnostic results are reported in Supplementary Table 4.

In addition, because no adjustment for multiple comparisons was applied. Given the number of outcome-specific, component-level, and sex-stratified tests, the corresponding *P* values should be regarded as nominal, and the findings should be interpreted as exploratory.

## 3 Results

### 3.1 Baseline characteristics of the participants

Among the 2,190 enrolled college students, the gender distribution was well-balanced, with males accounting for 50.05% (*n* =1,096) and females accounting for 49.95% (*n* = 1,094). Regarding physical fitness profiles, the proportions of participants stratified into the low, moderate, and high PFI categories were 25.02%, 49.95%, and 25.02%, respectively. According to the BMI classifications, the prevalence rates of underweight, normal weight, overweight, and obesity were 10.05%, 73.52%, 12.65%, and 3.79%, respectively. In terms of negative emotional symptoms, positive cases were detected in 1,491 participants (68.08%) for stress, 1,482 participants (67.67%) for anxiety, and 271 participants (12.37%) for depression.

Regarding lifestyle and physical activity covariates, the descriptive analysis revealed specific behavioral patterns: for MVPA engagement, the most frequently reported frequency was “2 days per week,” representing 28.04% of the cohort; for MSE, a notable proportion of students (25.34%) reported 0 days of engagement during the preceding week. In terms of autonomous exercise, 478 students engaged in unstructured physical activity 2 days per week, corresponding to a proportion of 21.83%. Conversely, non-participation was highly prevalent in organized sports, with 52.33% of the sample reporting 0 days of involvement. Digital lifestyle indicators demonstrated high levels of physical inactivity: more than half of the undergraduate population reported a daily screen time exceeding 3 h, and 69.59% of the participants accumulated more than 1 h of daily sedentary duration. The comprehensive baseline characteristics and behavioral distribution are detailed in Table 3.

**Table 3:** Basic situation of the sample.

Name	Options	Frequency	Percentage (%)	Name	Options.	Frequency	Percentage (%)
Gender	Female	1,094	49.95	MVPA	0 day	284	12.97
	Male	1,096	50.05		1 day	424	19.36
PFI	Low PFI	548	25.02		2 day	614	28.04
	Medium PFI	1,094	49.95		3 day	246	11.23
	High PFI	548	25.02		4 day	225	10.27
BMI	Emaciated	220	10.05		5 day	76	3.47
	Normal	1,610	73.52		6 day	321	14.66
	Overweight	277	12.65	MSE	0 day	555	25.34
	Obese	83	3.79		1 day	432	19.73
Stress	Normal	699	31.92		2 day	375	17.12
	Mild	464	21.19		3 day	329	15.02
	Moderate	310	14.16		4 day	133	6.07
	Severe	439	20.05		5 day	121	5.53
	Extremely severe	278	12.69		6 day	40	1.83
Anxiety	Normal	708	32.33		7 day	205	9.36
	Mild	295	13.47	Active exercise	0 day	332	15.16
	Moderate	556	25.39		1 day	449	20.50
	Severe	337	15.39		2 day	478	21.83
	Extremely severe	294	13.42		3 day	312	14.25
Depression	Normal	1,919	87.63		4 day	134	6.12
	Mild	132	6.03		5 day	485	22.15
	Moderate	62	2.83	Organized sports	0 day	1,146	52.33
	Severe	27	1.23		1 day	390	17.81
	Extremely severe	50	2.28		2 day	276	12.60
Duration of using electronic screens	No	42	1.92		3 day	137	6.26
	<1 h	77	3.52		4 day	56	2.56
	1–2 h	319	14.57		5 day	185	8.45
	2–3 h	592	27.03	Duration of meditation	No	210	9.59
	≧3 h	1,160	52.97		<1 h	456	20.82
					1–2 h	640	29.22
					2–3 h	445	20.32
					≧3 h	439	20.05

### 3.2 Univariate comparisons of depression, anxiety, and stress detection rates across different subgroups

Among the 2,190 surveyed college students, the positive detection rates / symptom-detection rates for stress, anxiety, and depression symptoms were 68.08% (*n* = 1,491), 67.67% (*n* = 1,482), and 12.37% (*n* = 271), respectively.

Pearson's chi-square tests showed that the prevalence of stress, anxiety, and depressive symptoms differed significantly across PFI, MVPA, MSE, autonomous exercise, and organized sports participation categories (χ^2^ values ranging from 12.28 to 1,098.56, all *P* ≤ 0.03; Table 4). The corresponding degrees of freedom and exact *P* values for each comparison are reported in Table 4.

**Table 4:** Comparison of the detection rates of depression, anxiety and stress symptoms among college students in different groups.

Group	Option	Number	Pressure				Anxiety				Depression			
			Detected	*χ2*	df	*p*	Detected	χ2	df	*P*	Detected	χ2	df	*p*
Gender	Female	1,094	734(67.09)	0.98	1	0.32	704(64.35)	11.02	1	<0.01	124(11.33)	2.18	1	0.14
	Male	1,096	757(69.07)				778(70.99)				147(13.41)			
PFI	Low PFI	548	511(93.25)	1,098.56	2	<0.01	481(87.77)	899.25	2	<0.01	135(24.64)	104.60	2	<0.01
	Medium PFI	1,094	918(83.91)				914(83.55)				102(9.32)			
	High PFI	548	62(11.31)				87(15.88)				34(6.20)			
BMI	Emaciated	220	161(73.18)	8.54	3	0.04	164(74.55)	22.41	3	<0.01	24(10.91)	2.43	3	0.49
	Normal	1,610	1,070(66.46)				1,047(65.03)				194(12.05)			
	Overweight	277	204(73.65)				215(77.62)				41(14.80)			
	Obese	83	56(67.47)				56(67.47)				12(14.46)			
MVPA	0 day	284	226(79.58)	151.34	6	<0.01	211(74.30)	31.67	6	<0.01	67(23.59)	78.06	6	<0.01
	1 day	424	331(78.07)				315(74.29)				79(18.63)			
	2 day	614	464(75.57)				418(68.08)				47(7.65)			
	3 day	246	160(65.04)				160(65.04)				11(4.47)			
	4 day	225	113(50.22)				143(63.56)				29(12.89)			
	5 day	76	32(42.11)				50(65.79)				8(10.53)			
	6 day	321	165(51.40)				185(57.63)				30(9.35)			
MSE	0 day	555	424(76.40)	76.53	7	<0.01	415(74.77)	33.03	7	<0.01	105(18.92)	40.39	7	<0.01
	1 day	432	323(74.77)				306(70.83)				60(13.89)			
	2 day	375	254(67.73)				245(65.33)				36(9.60)			
	3 day	329	221(67.17)				219(66.57)				25(7.60)			
	4 day	133	79(59.40)				82(61.65)				8(6.02)			
	5 day	121	62(51.24)				73(60.33)				9(7.44)			
	6 day	40	18(45.00)				20(50.00)				4(10.00)			
	7 day	205	110(53.66)				122(59.51)				24(11.71)			
Active exercise	0 day	332	247(74.40)	69.17	5	<0.01	244(73.49)	46.79	5	<0.01	68(20.48)	40.51	5	<0.01
	1 day	449	340(75.72)				323(71.94)				72(16.04)			
	2 day	478	354(74.06)				352(73.64)				49(10.25)			
	3 day	312	203(65.06)				207(66.35)				25(8.01)			
	4 day	134	79(58.96)				81(60.45)				8(5.97)			
	5 day	485	268(55.26)				275(56.70)				49(10.10)			
Organized sports	0 day	1,146	841(73.39)	74.09	5	<0.01	822(71.73)	40.03	5	<0.01	165(14.40)	12.28	5	0.03
	1 day	390	268(68.72)				264(67.69)				45(11.54)			
	2 day	276	189(68.48)				188(68.12)				27(9.78)			
	3 day	137	82(59.85)				82(59.85)				15(10.95)			
	4 day	56	29(51.79)				33(58.93)				2(3.57)			
	5 day	185	82(44.32)				93(50.27)				17(9.19)			
Duration of using electronic screens	No	42	26(61.90)	2.36	4	0.67	27(64.29)	5.78	4	0.22	7(16.67)	6.62	4	0.16
	<1 h	77	54(70.13)				54(70.13)				8(10.39)			
	1–2 h	319	212(66.46)				209(65.52)				34(10.66)			
	2–3 h	592	396(66.89)				383(64.70)				61(10.30)			
	≧3 h	1,160	803(69.22)				809(69.74)				161(13.88)			
Duration of meditation	No	210	142(67.62)	6.60	4	0.16	141(67.14)	9.72	4	0.05	33(15.71)	13.44	4	0.01
	<1 h	456	322(70.61)				322(70.61)				69(15.13)			
	1–2 h	640	414(64.69)				413(64.53)				67(10.47)			
	2–3 h	445	301(67.64)				290(65.17)				40(8.99)			
	≧3 h	439	312(71.07)				316(71.98)				62(14.12)			

Data are presented as *n* (%), unless otherwise indicated. Percentages were calculated within each subgroup. χ^2^ statistics were obtained using Pearson's chi-square tests, with the corresponding degrees of freedom shown in parentheses. All *P* values are two-sided and unadjusted for multiple comparisons. *P* values are reported to four decimal places; values below 0.01 are presented as *P* < 0.01.

Across BMI categories, significant differences were observed for stress [χ^2^(3) = 8.54, *P* = 0.0360] and anxiety [χ^2^(3) = 22.41, *P* < 0.01], but not for depression [χ^2^(3) = 2.43, *P* = 0.49]. A significant sex difference was observed for anxiety [χ^2^(1) = 11.02, *P* < 0.01], whereas no significant sex differences were found for stress [χ^2^(1) = 0.98, *P* = 0.32] or depression [χ^2^(1) = 2.18, *P* = 0.14].

Daily sedentary time was significantly associated with anxiety [χ^2^(4) = 9.72, *P* = 0.05] and depression [χ^2^(4) = 13.44, *P* < 0.01], but not with stress [χ^2^(4) = 6.60, *P* = 0.16]. Daily screen time was not significantly associated with stress [χ^2^(4) = 2.36, *P* = 0.67], anxiety [χ^2^(4) = 5.78, *P* = 0.22], or depression [χ^2^(4) = 6.62, *P* = 0.16]. The complete univariate comparison results are presented in Table 2.

### 3.3 Multivariable logistic regression analysis of individual physical fitness metrics and negative emotional symptoms

The multivariable Logistic regression analysis revealed pronounced gender-specific variations in the independent associations between individual physical fitness metrics and the risks of manifesting stress, anxiety, and depressive symptoms (detailed in Table 1).

#### 3.3.1 Female students

Stress: Higher performance levels in vital capacity, the standing long jump, muscular strength, and the sit-and-reach test were significantly associated with a reduced risk of stress symptom detection. Conversely, prolonged completion times in the 50-meter sprint and endurance runs (indicative of poorer functional capacity) were explicitly correlated with an elevated risk of stress symptoms.
Anxiety: Incremental increases in vital capacity, standing long jump distance, muscular strength, and flexibility scores were protective factors against anxiety, whereas prolonged durations in the endurance run independently predicted an increased risk of anxiety detection.
Depression: Notably, none of the individual physical fitness metrics reached statistical significance in their associations with the risk of depressive symptom detection among female students (all *P* > 0.05).

#### 3.3.2 Male students

Stress & Anxiety: Enhanced performance in vital capacity, muscular strength, and the sit-and-reach test was robustly associated with decreased odds of both stress and anxiety detection. In contrast, prolonged execution times in the 50-meter sprint and endurance runs significantly amplified the risk of both stress and anxiety manifestation. Additionally, superior standing long jump performance was uniquely associated with a reduced risk of anxiety; however, its protective association with stress symptoms did not reach statistical significance (*P* > 0.05).
Depression: In male students, longer 50-m sprint time (OR = 1.35, 95% CI 1.06–1.71; *P* = 0.010) and longer endurance-run time (OR = 1.40, 95% CI 1.14–1.73; *P* < 0.001) were associated with higher odds of depressive symptoms. The remaining physical-fitness components—vital capacity, standing long jump, muscular strength, and sit-and-reach—were not statistically significant.

### 3.4 Multivariable logistic regression analysis of global PFI and negative emotional symptoms

Cross-tabulation showed marked differences in outcome distribution across PFI categories. In the low, moderate, and high PFI groups, respectively, positive cases numbered 511, 918, and 62 for stress; 481, 914, and 87 for anxiety; and 135, 102, and 34 for depression (Supplementary Table 1). The small number of positive stress and anxiety cases in the high-PFI group motivated penalized and continuous-exposure sensitivity analyses.

In the pooled sample, conventional adjusted logistic regression showed lower odds in the moderate PFI group for stress (OR = 0.346, 95% CI 0.230–0.518), anxiety (OR = 0.701, 95% CI 0.511–0.962), and depression (OR = 0.329, 95% CI 0.245–0.441). Corresponding estimates for high PFI were 0.004 (95% CI 0.003–0.007), 0.023 (95% CI 0.016–0.033), and 0.221 (95% CI 0.146–0.334), respectively (Table 2). The exceptionally small high-PFI estimates for stress and anxiety should not be interpreted as precise causal effect sizes.

Firth penalized estimates were directionally consistent with lower odds in the high PFI category for all outcomes in female and male students (all *P* < 0.001). For moderate PFI, however, the associations with stress in female students (OR = 0.623, 95% CI 0.366–1.031; *P* = 0.066) and anxiety in male students (OR = 0.802, 95% CI 0.451–1.381; *P* = 0.432) were not statistically significant; the remaining moderate-PFI comparisons were significant (Supplementary Table 2). Thus, the verified results do not support a uniformly significant stepwise pattern in every sex-outcome stratum.

When PFI was modeled continuously, each 1-SD increase was associated with lower odds of stress (OR = 0.120, 95% CI 0.098–0.146), anxiety (OR = 0.217, 95% CI 0.186–0.253), and depression (OR = 0.602, 95% CI 0.525–0.689) in the pooled sample; all sex-stratified continuous associations were also significant (all *P* < 0.001; Supplementary Table 3). All models converged, and no substantial multicollinearity was detected. However, several Hosmer–Lemeshow tests suggested possible calibration limitations. Therefore, the consistency in the direction of association across conventional, Firth-penalized, and continuous-PFI models is more informative than the exact magnitude of the extreme categorical ORs.

## 4 Discussion

### 4.1 Prevalence of negative emotional symptoms and the protective role of physical fitness metrics

These symptom-detection rates require comparison with external evidence. The anxiety detection rate in this sample (67.67%) was substantially higher than the global pooled estimate of 39.0% (95% CI 34.6%−43.4%) reported for college students, whereas the depression rate (12.37%) was lower than the corresponding pooled estimate of 33.6% (95% CI 29.3%−37.8%) (Li et al., 2022). That meta-analysis did not provide a directly comparable pooled stress estimate. Accordingly, the present stress and anxiety figures should not be generalized as clinical prevalence without considering instrument, threshold, sampling, and timing differences. This observation implies that the psychological distress burden among contemporary undergraduate populations remains profoundly elevated, a finding that is highly congruent with the global reports by Wang X et al., who documented that over 70% of surveyed international college students experienced marked escalations in anxiety and stress driven by academic pressures and environmental uncertainties (Gao et al., 2020; Wang et al., 2020).

A key measurement consideration is that DASS-21 is a symptom-screening instrument rather than a diagnostic interview. Defining positive status as mild-or-higher (stress ≥15; anxiety ≥8) deliberately captures transient and subclinical symptoms, which can yield higher detection rates than studies using moderate/severe thresholds or clinical diagnoses. The figures should therefore be described as symptom-detection rates, not disorder prevalence.

Data were collected between December 2025 and January 2026, overlapping with end-of-term academic and physical-fitness assessments. This context may have contributed to short-term stress and anxiety, but the present cross-sectional data cannot quantify that contribution. The clustered sample of second-year students from Chongqing may also differ from samples included in international meta-analyses (Wang et al., 2016).

Academic competition, employment concerns, digital lifestyles, and social adaptation are plausible contextual stressors (Zhang et al., 2025c; Elhai et al., 2017), but they were not all measured in this study. They should therefore be treated as possible explanations rather than demonstrated causes of the high stress and anxiety detection rates.

The cornerstone innovation of the present study lies in the revelation that the robust, independent inverse associations between the global PFI and the risks of manifesting three distinct negative emotional symptoms persisted, even after a comprehensive suite of behavioral exercise variables (MVPA, MSE, autonomous exercise, and organized sports participation) and critical lifestyle confounders (screen time, sedentary duration, and BMI classifications) were systematically adjusted for. Compared with the low PFI tier, individuals in the moderate and high PFI groups exhibited pronounced reductions in psychological distress risks. This crucial empirical insight strongly corroborates the ongoing paradigm shift currently advocated within the international scientific community—namely, the conceptual transition from behavioral engagement to physiological function in mental health epidemiology (Dishman et al., 2015; Kandola et al., 2019; Schuch et al., 2018). However, this study several categorical ORs for the high-PFI group were extremely small, positive outcome cells were sparse, and some Hosmer–Lemeshow tests indicated possible calibration limitations. Although the conventional, Firth-penalized, and continuous-PFI analyses were directionally consistent, the exact magnitudes of the extreme categorical ORs should be interpreted cautiously and should not be regarded as precise causal effect estimates. In addition, because Tables 1, 2 represent separate association models rather than formal comparisons of predictive performance, the results do not establish that PFI predicts mental health outcomes better than its individual components or exercise behavior. PFI should therefore be viewed as a complementary composite indicator whose predictive and clinical utility requires prospective external validation.

PFI provides a complementary composite representation of cardiorespiratory, neuromuscular, and flexibility domains. Prospective validation and direct model-comparison studies would be needed to establish incremental predictive value. While a vast body of historical literature has remained predominantly anchored in subjective, self-reported behavioral indices (i.e., mapping “how long or how frequently” an individual exercises), our findings provide compelling evidence that an individual's objectively verified, accrued PFI possesses significantly superior predictive and protective power for mental health than mere exercise frequency or behavioral participation alone (Kandola et al., 2020). This disparity can be elucidated by the fact that the comprehensive Physical Fitness Index integrates multi-systemic physiological capacities, including cardiorespiratory efficacy and neuromuscular adaptation. Consequently, rather than being a mere reflection of acute lifestyle habits, the PFI represents the ultimate phenotypic physiological manifestation of a complex, long-term interaction among an individual's genetic endowment, habitual physical behaviors, and environmental exposures. It essentially serves as a robust, resilient somatic buffer that shields the central nervous system against external psychological stressors.

### 4.2 Mechanistic insights: biological, behavioral, and psychosocial pathways

From a mechanistic perspective, the synergistic protective effects of superior global PFI against negative emotional symptoms can be robustly interpreted through the theoretical lenses of the “bio psychosocial model of exercise” and the “cross-stressor adaptation (CSA) hypothesis.”

The Biological Dimension (Neuroendocrine Remodeling): Sustained optimal physical fitness induces a profound remodeling of the central nervous and endocrine microenvironments. Physically fit individuals exhibit enhanced neurotrophic support within the central nervous system, characterized by the upregulated synthesis and release of brain-derived neurotrophic factor (*BDNF*), alongside optimized neurochemical pathways for dopamine and serotonin (5−*HT*) (Cotman et al., 2007). Concurrently, the CSA hypothesis posits that high-PFI individuals possess a more resilient and tightly regulated hypothalamic-pituitary-adrenal (HPA) axis. When exposed to psychological stressors, these individuals exhibit a blunted, adaptive cortisol response and a downstream down regulation of systemic pro-inflammatory cytokines—such as interleukin-6 (*IF*−6) and tumor necrosis factor-alpha (*TNF*−∝)—thereby biophysically obstructing the pathophysiological cascade of anxiety and depression (Schuch et al., 2019).

The Behavioral and Psychological Dimension (Sleep Architecture and Recovery): High cardiorespiratory and muscular fitness are naturally coupled with optimized sleep architecture, characterized by higher sleep efficiency and a greater proportion of slow-wave (deep) sleep. This superior sleep quality serves as a direct homeostatic mechanism that repairs accumulated daytime neural fatigue and mitigates emotional depletion (Kredlow et al., 2015).

The Social Dimension (Identity Reconstruction and Social Capital): Objective physical competencies directly enhance individual “body image,” which in turn boosts physical self-efficacy and core self-esteem. Furthermore, higher-fitness individuals naturally garner greater opportunities for prosocial interaction and peer support, constructing a rich reservoir of psychosocial resources that effectively buffers academic and adaptive stressors (Fox, 2003). It should be pointed out that although this study provides theoretical explanations for these mechanisms, it does not directly test the statistical mediation effect. In this regard, the results of this study are consistent with recent evidence formally testing such mediation paths: Zhao et al. (2025) confirmed in a sample of China college students that self-esteem significantly mediates the relationship between physical activity and depression and anxiety symptoms, providing direct empirical support for the “identity reconstruction” hypothesis proposed in this paper.

### 4.3 The dominance of cardiorespiratory fitness and the novel contribution of somatic flexibility

When dissecting individual components via multivariable Logistic regression, speed (50-meter sprint) and cardiorespiratory endurance (800/1,000-meter runs) emerged as the most prominent predictors of negative emotional symptoms. Within sports sciences, these two performance metrics serve as core field-based proxies for cardiorespiratory fitness (CRF). Our study demonstrated that, irrespective of gender, prolonged completion times in these running events (indicative of depleted CRF) significantly elevated the risks of stress and anxiety detection. This empirical pattern highlights CRF as a highly sensitive fitness domain within the brain's central emotion-regulation networks. Suboptimal CRF not only signifies compromised aerobic oxidative capacity and restricted fatigue reserves, but also triggers a cascade of diminished exercise self-confidence and the chronic somatic accumulation of daytime fatigue, ultimately rendering the individual's psychological defense mechanisms vulnerable to collapse under chronic academic strain (Kandola et al., 2019; Dishman et al., 2015).

Concurrently, vital capacity, muscular strength (pull-ups/sit-ups), and flexibility (sit-and-reach) displayed consistent protective associations. While vital capacity reflects respiratory system reserve and oxygen kinetics, muscular strength indicates the functional capacity of the neuromuscular system—a finding that strongly supports the burgeoning global clinical paradigm favoring resistance training as a primary non-pharmacological intervention for psychiatric disorders (Gordon et al., 2018).

Notably, the significant association observed between flexibility (the sit-and-reach test)—traditionally regarded as a rudimentary baseline component—and the mitigation of stress and anxiety offers profound academic insights. This phenomenon can be elegantly elucidated by the somatization activation theory. Chronic psychological distress and anxiety frequently manifest physically as persistent, generalized neuromuscular tension (somatization symptoms). Individuals possessing superior musculoskeletal flexibility inherently exhibit lower resting muscle tension. This structural laxity is hypothesized to downregulate central anxiety alarms via the counter-activation of the parasympathetic nervous system, thereby enhancing subjective somatic comfort and restoring emotional homeostasis (Behm et al., 2026).

### 4.4 Gender-specific divergence in the fitness-mental health nexus

The sex-stratified multivariable Logistic regression models unveiled a profound gender-specific divergence in the behavioral and psychological patterns linking physical fitness metrics to negative emotional symptoms.

**(1) The performance-driven fitness sensitivity mechanism in male students**

In male students, depressive symptoms exhibited a highly localized sensitivity to performance-based, explosive physical metrics, specifically the 50-meter sprint and the 1,000-meter run, where sub-optimal performance significantly exacerbated the risk of depression. This empirical phenomenon is deeply anchored in traditional gender role expectations and competitive achievement motivation. Within contemporary campus subcultures, speed and cardiorespiratory endurance are rarely interpreted as mere physiological indicators; rather, they are heavily heavily operationalized as socially constructed markers of “athletic prowess,” “dominance,” and “masculine competence” (Sharp et al., 2024). Consequently, a deficit in these high-visibility, performance-driven fitness categories can easily induce a state of chronic exercise-related inferiority, body dissatisfaction, and perceived physical incompetence among male undergraduates, ultimately precipitating the clinical cascade into anhedonia and depressive manifestations.

**(2) The multi-dimensional holistic protective architecture in female students**

Conversely, female students manifested a distinct, multi-dimensional holistic protective pattern. Although no single, isolated physical fitness metric reached statistical significance in predicting depressive symptoms among females, their global aggregated PFI demonstrated an exceptionally robust, protective threshold against all three negative emotional domains. This striking contrast implies that the psychological resilience of female college students does not depend on singular, high-performance athletic indices, but is rather sustained by a synergistic, multi-systemic baseline health matrix that encapsulates cardiorespiratory reserve, muscular capacity, and somatic flexibility in unison (Xu et al., 2026). For the female cohort, psychological wellbeing appears to be anchored in the overall harmony of the somatic profile rather than isolated competitive excellence, a finding that underscores the necessity of adopting a holistic health-quality paradigm—rather than a performance-centric sports model—when developing targeted mental health interventions for female college students. Similarly, Levante et al. (2024) reported in a sample of female college students that the protective effect of physical exercise habits on psychological distress is mediated by multiple physiological and psychosocial factors, a finding that is highly consistent with the “holistic” female pattern observed in this study and suggests that future research should explicitly test multiple mediation models rather than just relying on direct associations.

### 4.5 Behavioral nuances of digital lifestyles and targeted practical implications

Within the domain of digital lifestyle indicators, the intriguing juxtaposition wherein “screen time exhibited no statistical correlation, while sedentary duration was significantly tied to anxiety and depression” aligns seamlessly with cutting-edge behavioral epidemiology. Non-academic screen engagement operates as a double-edged sword (Choukas-Bradley et al., 2023; Van de Casteele et al., 2026). While screen use inevitably triggers a “displacement effect” that reduces physical activity time, the intrinsic nature of the content—ranging from immersive social networking and hyper-interactive gaming to short-form entertainment—frequently offers a mechanism for hedonic escape or psychological compensation. This digital coping strategy may dilute or neutralize its potential psychopathological side effects (Choukas-Bradley et al., 2023; Van de Casteele et al., 2026). Conversely, prolonged sedentary behavior constitutes an independent risk factor distinct from physical inactivity. An absolute, prolonged state of low energy expenditure severely suppresses baseline metabolic kinetics, dampens central nervous system arousal, and accelerates the somatic accumulation of fatigue, thereby directly biophysically catalyzing the onset of anxious and depressive symptoms (Subiron-Valera et al., 2023).

Grounded in these empirical findings, the paradigm for campus mental health promotion must evolve beyond the conventional, unilineal framework of passive psychological counseling. Instead, universities should pioneer an integrated ecosystem that harmonizes objective physical fitness cultivation with proactive psychological support. This actionable strategy can be operationalized across two key dimensions:

**(1) Establishing a PFI-driven stratified screening and early-warning system**

Higher education institutions should systematically harness the infrastructure of annual mandatory national student physical fitness testing, but the sample-relative PFI categories should not be used as stand-alone clinical or campus early-warning thresholds. Universities could consider objective fitness information as one component of broader, voluntary health-promotion assessment, alongside validated mental-health screening and professional evaluation.

**(2) Implementing precision, gender-stratified, and phase-specific exercise prescriptions**

Behavioral Rehabilitation for the Low-PFI Cohort: To safeguard the psychological safety of low-fitness individuals, competitive, high-intensity contact sports should be circumvented initially. Interventions should commence with brisk walking and low-intensity circuit resistance training, with the primary pedagogical objective of reconstructing their exercise self-confidence and physical self-efficacy.

Performance-Driven Aerobic Interventions for Male Students: Given the specific fitness sensitivity identified in males, prescriptions should intentionally integrate high-energy endurance stimuli, such as Fartlek running, high-intensity interval training (HIIT), and comprehensive physical conditioning drills, to directly dismantle their speed and cardiorespiratory endurance deficits.

Core and Flexibility Matrix Optimization for Female Students: Aligning with the holistic fitness matrix crucial to females, interventions should prioritize Pilates, core circuit resistance training, and artistic flexibility extension modalities, thereby globally enhancing their baseline somatic health quality and subjective physical comfort.

### 4.6 Methodological limitations and future research directions

Despite its strengths, several inherent methodological limitations of the present study must be acknowledged:

First, the cross-sectional design prevents determination of temporal order or causality. Reverse causation is plausible: pre-existing stress, anxiety, or depressive symptoms may reduce exercise motivation and participation, disrupt sleep or nutrition, and thereby contribute to poorer physical-fitness performance. Bidirectional pathways are also possible; longitudinal and experimental designs are required to distinguish them.

Second, although biological, sleep-related, and psychosocial pathways were discussed, these potential mediators were not measured or formally tested. The mechanistic discussion is therefore hypothesis-generating, and residual confounding remains possible.

Third, PFI categories were defined using sex-specific P25 and P75 values from this sample. They represent relative ranks rather than externally validated clinical thresholds, and the same numerical PFI could be categorized differently across populations or sexes. This limits direct generalizability and supports reporting continuous PFI alongside categories.

Fourth, DASS-21 measures symptoms during the preceding week and is not a diagnostic instrument. The mild-or-higher thresholds increase sensitivity but reduce comparability with studies using clinical interviews or more stringent cutoffs.

Fifth, although the study included participants from 73 universities, all participating institutions were located within Chongqing Municipality. Chongqing has socioeconomic, cultural, geographic, and educational characteristics that may differ from those of other regions of China. Accordingly, the present sample should not be considered nationally representative, and the observed symptom prevalence estimates and associations between PFI and negative emotional symptoms may not be directly generalizable to college students in other provinces or regions.

Future investigations should use multicenter longitudinal cohorts and randomized trials to evaluate temporal ordering, dose-response patterns, mediation, and causal effects. Wearable measures and repeated psychological assessments could strengthen exposure measurement and reduce recall bias. External validation of PFI parameterization and any proposed cutoffs is also needed before clinical or campus implementation.

## Data Availability

The raw data supporting the conclusions of this article will be made available by the authors, without undue reservation.
