# Supplementary material for: Association between college students 'physical fitness index and negative emotional symptoms: an empirical study based on college students in Chongqing City, China
Source: Front Psychol. 2026 Sep 24;17:1927005. doi: 10.3389/fpsyg.2026.1927005 (PMC13645542; doi:10.3389/fpsyg.2026.1927005)
Supplement: Supplementary file 1 [file Data_Sheet_1.pdf]

Supplementary Table S1 Distribution of DASS-21 symptom status across PFI categories

Outcome	PFI category	N	Negative, n (%)	Positive, n (%)	Positive threshold
Stress	Low PFI	548	37 (6.8)	511 (93.2)	≥15
	Moderate PFI	1094	176 (16.1)	918 (83.9)	≥15
	High PFI	548	486 (88.7)	62 (11.3)	≥15
	Total	2190	699 (31.9)	1491 (68.1)	≥15
Anxiety	Low PFI	548	67 (12.2)	481 (87.8)	≥8
	Moderate PFI	1094	180 (16.5)	914 (83.5)	≥8
	High PFI	548	461 (84.1)	87 (15.9)	≥8
	Total	2190	708 (32.3)	1482 (67.7)	≥8
Depression	Low PFI	548	413 (75.4)	135 (24.6)	≥10
	Moderate PFI	1094	992 (90.7)	102 (9.3)	≥10
	High PFI	548	514 (93.8)	34 (6.2)	≥10
	Total	2190	1919 (87.6)	271 (12.4)	≥10

Note: Percentages were calculated within each population – PFI stratum. PFI categories were based on sex-specific sample percentiles: low PFI <P25, moderate PFI P25 – P75, and high PFI >P75. DASS-21 subscale scores were multiplied by two, and positive status was defined as mild-or-higher symptoms. No marginal outcome cell contained zero cases.

Supplementary Table S2 Firth penalized logistic regression for categorical PFI and DASS-21 symptom outcomes

Population	Outcome	PFI category	OR	95% CI	P	N	Events
Overall	Stress	Low PFI (reference)	1.000	Reference	—	2190	1491
		Moderate PFI	0.356	0.236–0.526	<0.001	2190	1491
		High PFI	0.005	0.003–0.008	<0.001	2190	1491
	Anxiety	Low PFI (reference)	1.000	Reference	—	2190	1482
		Moderate PFI	0.707	0.514–0.963	.028	2190	1482
		High PFI	0.025	0.017–0.035	<0.001	2190	1482
	Depression	Low PFI (reference)	1.000	Reference	—	2190	271
		Moderate PFI	0.335	0.250–0.448	<0.001	2190	271
		High PFI	0.228	0.150–0.339	<0.001	2190	271
Female	Stress	Low PFI (reference)	1.000	Reference	—	1094	734
		Moderate PFI	0.623	0.366–1.031	.066	1094	734
		High PFI	0.003	0.001–0.006	<0.001	1094	734
	Anxiety	Low PFI (reference)	1.000	Reference	—	1094	704
		Moderate PFI	0.676	0.456–0.987	.043	1094	704
		High PFI	0.054	0.034–0.083	<0.001	1094	704
	Depression	Low PFI (reference)	1.000	Reference	—	1094	124
		Moderate PFI	0.509	0.330–0.784	.002	1094	124
		High PFI	0.405	0.230–0.694	<0.001	1094	124
Male	Stress	Low PFI (reference)	1.000	Reference	—	1096	757
		Moderate PFI	0.154	0.076–0.291	<0.001	1096	757
		High PFI	0.003	0.001–0.007	<0.001	1096	757
	Anxiety	Low PFI (reference)	1.000	Reference	—	1096	778
		Moderate PFI	0.802	0.451–1.381	.432	1096	778
		High PFI	0.008	0.004–0.016	<0.001	1096	778
	Depression	Low PFI (reference)	1.000	Reference	—	1096	147
		Moderate PFI	0.236	0.155–0.356	<0.001	1096	147
		High PFI	0.135	0.069–0.247	<0.001	1096	147

Note: OR, odds ratio; CI, profile penalized-likelihood confidence interval. Firth models used Jeffreys-prior penalization and likelihood-ratio P values. Overall models adjusted for sex, BMI category, MVPA days/week, MSE days/week, autonomous exercise frequency, organized sports participation, screen-time category, and sedentary-time category; sex-stratified models omitted sex. Reference levels were low PFI, female sex, normal BMI, 0 autonomous exercise sessions/week, 0 organized sports sessions/week, no screen time, and no sedentary time. MVPA and MSE were entered linearly per day/week. All nine models converged.

Supplementary Table S3 Sensitivity analysis using continuous standardized PFI (per 1-SD increase)

Population	Outcome	Exposure	OR	95% CI	P	N (events)
Overall	Stress	PFI per 1 SD	0.120	0.098–0.146	<0.001	2190 (1491)
	Anxiety	PFI per 1 SD	0.217	0.186–0.253	<0.001	2190 (1482)
	Depression	PFI per 1 SD	0.602	0.525–0.689	<0.001	2190 (271)
Female	Stress	PFI per 1 SD	0.100	0.075–0.134	<0.001	1094 (734)
	Anxiety	PFI per 1 SD	0.307	0.256–0.370	<0.001	1094 (704)
	Depression	PFI per 1 SD	0.686	0.567–0.829	<0.001	1094 (124)
Male	Stress	PFI per 1 SD	0.112	0.083–0.152	<0.001	1096 (757)
	Anxiety	PFI per 1 SD	0.111	0.083–0.149	<0.001	1096 (778)
	Depression	PFI per 1 SD	0.513	0.418–0.631	<0.001	1096 (147)

Note: PFI was standardized using the overall sample SD (3.5003 units). Models were conventional maximum-likelihood logistic regressions with Wald 95% CIs and two-sided P values. Covariate adjustment and reference levels were the same as in Supplementary Table S2.

Supplementary Table S4 Diagnostics for conventional categorical-PFI logistic regression models

Population	Outcome	N	Events	Converged	H–L χ^2 (df)	H–L P	Nagelkerke R^2	Max adj. GVIF
Overall	Stress	2190	1491	Yes	31.41 (8)	<0.001	0.649	1.517
	Anxiety	2190	1482	Yes	36.02 (8)	<0.001	0.505	1.507
	Depression	2190	271	Yes	36.19 (8)	<0.001	0.123	1.515
Female	Stress	1094	734	Yes	14.97 (8)	.060	0.704	1.587
	Anxiety	1094	704	Yes	10.64 (8)	.223	0.371	1.607
	Depression	1094	124	Yes	11.04 (8)	.200	0.067	1.619
Male	Stress	1096	757	Yes	18.30 (8)	.019	0.670	1.618
	Anxiety	1096	778	Yes	15.73 (8)	.046	0.675	1.461
	Depression	1096	147	Yes	43.70 (8)	<0.001	0.254	1.444

Note: H－L, Hosmer－Lemeshow test using 10 approximately equal-sized groups; GVIF, generalized variance inflation factor. Max adj. GVIF is the maximum GVIF^(1/[2 × df]) across model terms. Significant H－L tests in the pooled and male models indicate possible calibration limitations; these results, together with sparse outcome cells, motivated the Firth and continuous-PFI sensitivity analyses.
